# Quantitative analysis of diffraction by liquids using a pink-spectrum X-ray source

**DOI:** 10.1107/S1600577522004076

**Published:** 2022-05-16

**Authors:** Saransh Singh, Amy L. Coleman, Shuai Zhang, Federica Coppari, Martin G. Gorman, Raymond F. Smith, Jon H. Eggert, Richard Briggs, Dayne E. Fratanduono

**Affiliations:** a Lawrence Livermore National Laboratory, Computational Engineering Division, Livermore, CA 94511, USA; b Laboratory for Laser Energetics, University of Rochester, Rochester, NY 14623, USA

**Keywords:** pink beam, liquid scattering, shock compression, Dynamic Compression Sector

## Abstract

A new approach for performing quantitative structure-factor analysis and density measurements of liquids using X-ray diffraction with a pink-spectrum X-ray source is described. The methodology corrects for the pink beam effect by performing a Taylor series expansion of the diffraction signal. The mean density, background scale factor, peak X-ray energy about which the expansion is performed, and the cutoff radius for density measurement are estimated using the derivative-free optimization scheme. The formalism is demonstrated for a simulated radial distribution function for tin. Finally, the proposed methodology is applied to experimental data on shock compressed tin recorded at the Dynamic Compression Sector at the Advanced Photon Source, with derived densities comparing favorably with other experimental results and the equations of state of tin.

## Abstract 2

A new approach to compute structure factors, radial distribution function and mean density from liquid scattering data in a pink X-ray source is described.

## 1. Introduction

Over the last two decades marked improvements have been made to both the experimental and analytical techniques associated with the study of dense liquid states using X-ray diffraction and static high-pressure techniques (Eggert *et al.*, 2002 ▸; Morard *et al.*, 2014 ▸). Typically, studies of high-pressure liquids (*P* < 100 GPa) have relied on the diamond anvil cell (DAC) apparatus, which consists of two opposing diamond anvils that compress a sample (surrounded by a pressure-transmitting medium) in a metallic chamber compressed between the two anvils. While much important research has been conducted using static compression techniques at relatively low pressures, *e.g.* the observation of first-order liquid–liquid phase transitions (Katayama *et al.*, 2000 ▸; Soper & Benmore, 2008 ▸), and the critical point in sulfur near 2.0 GPa (Henry *et al.*, 2020 ▸), there is an inherent limit to the accessible pressure states imposed by the strength of the diamonds in the cell, as well as an upper temperature limit imposed by the physical geometry of the DAC apparatus (Anzellini & Boccato, 2020 ▸). Additionally, the thick diamonds used in the DAC setup are known to make a significant contribution to the X-ray diffraction signal collected during high-temperature, high-pressure liquid experiments, along with scattering contributions from the surrounding medium. The removal of these parasitic features can be non-trivial and is essential for the proper analysis of the X-ray scattering from the material of interest (Eggert *et al.*, 2002 ▸; Morard *et al.*, 2014 ▸). The recent implementation of Soller slits has facilitated the collection of high-quality diffraction data from low-*Z* liquids at pressures just over 1 Mbar (Weck *et al.*, 2017 ▸); however, small sample sizes and the practical difficulties of using DACs under these conditions mean that it has so far been impossible to access the multi-megabar regime for these types of experiments (*P* > 200 GPa).

**Figure 1:**
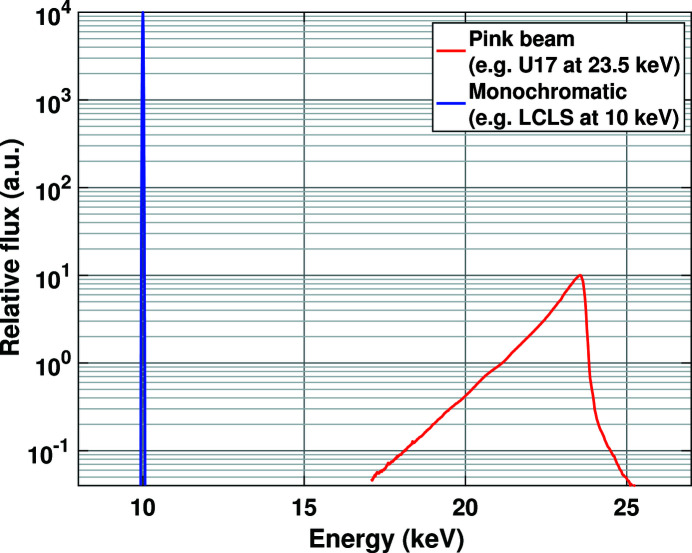
Plot of representative (not measured) X-ray photon energy versus flux at the Linac Coherent Light Source (blue) and U17 flux measured at the Dynamic Compression Sector (red).

The advent of fourth-generation light sources such as the Linac Coherent Light Source (LCLS) presents a new method of probing dense liquid states as generated through laser-driven dynamic compression experiments. The short timescales of such experiments require highly brilliant X-rays in order to obtain single-exposure diffraction data of high enough quality to perform quantitative analysis of liquid scattering data (Briggs *et al.*, 2019*a* ▸). These shock-compression experiments grant access to pressure states up to several Mbar, vastly broadening the scope of the study of dense liquids. Liquid diffraction data have been successfully collected at LCLS (Briggs *et al.*, 2017*a* ▸; Gorman *et al.*, 2018 ▸; Coleman *et al.*, 2019 ▸); however, the detector coverage and the accessible momentum transfer or *q*-range over which diffraction data may be obtained can be limited. Recent years have seen the addition of such laser systems to synchrotron beamlines, meaning that laser-driven dynamic compression experiments can now also be conducted at facilities that have produced high-quality liquid diffraction data from DACs over the previous two decades. These facilities have the capability to collect diffraction on sub-nanosecond timescales as well as providing detector coverage from a single-panel detector for full azimuthal coverage making them excellent candidates for probing dynamically compressed liquids. The dynamic compression sector (DCS) at the Advanced Photon Source (APS), having recently installed a 100 J laser system, is one such facility that affords users the opportunity to dynamically compress samples (Wang *et al.*, 2019 ▸). This beamline is equipped with a U17 undulator, providing a non-monochromatic X-ray source, commonly referred to as a pink beam. A representative X-ray photon flux versus energy curve for the X-ray free-electron source at LCLS and the as-measured spectral flux from the U17 undulator at APS is shown in Fig. 1 ▸. The full width at half-maxima (FWHM) for the energy–flux distribution at DCS is ∼0.785 keV, which is a 3.3% spread around the energy of peak flux. The FWHM at LCLS is ∼0.2%. It is also worth noting that the relative flux at LCLS-II is about three orders of magnitude brighter compared with DCS.

The scattering of X-rays from a liquid sample produces a broad diffuse signal that contains information on the short range order of the atoms. The shape and location of the broad liquid peaks are affected by the X-ray source type. A monochromatic X-ray beam (with bandwidth Δ*E*/*E* 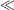 1%) will typically produce peaks at a scattering angle defined by the average atomic positions. However, if the X-ray source is a pink-beam source, with an intensity profile characterized by a sharp Gaussian fall-off at higher energies and an exponential tail to lower energies with Δ*E*/*E* ≃ 3% (as is the case for the U17 undulator at DCS), then there is an artificial shift of the liquid peak locations to higher scattering angles and an asymmetry in the peak profile (Bratos *et al.*, 2014 ▸). Analysis of the liquid scattering intensities as a function of momentum transfer, *q* (where *q* = 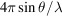 , θ is the scattering angle, and λ is the X-ray wavelength), provides the liquid structure factor, which in conjunction with the mean density can be used to determine the radial distribution function (Kaplow *et al.*, 1965 ▸; Eggert *et al.*, 2002 ▸).

In this paper, we present a new approach to quantitative structure factor and density determination in liquid diffraction data obtained using a pink X-ray source. The implementation of a Taylor series expansion of the spectra can be used to account for the artifacts introduced by the pink X-ray beam. Furthermore, the corrected spectra can be used in an optimization procedure to determine the density of the compressed liquid state. We will only focus on the case of monatomic liquid in this manuscript. The extension to the case of polyatomic liquids, although tedious, follows the same general steps. The *Methods* [Sec sec2] section briefly describes the scattering of X-rays by monatomic liquids. Diffraction signal modulation in the presence of a pink X-ray beam is also described. A method based on the Taylor series expansion of the coherent diffraction intensity to correct for this effect is presented. Finally, we describe the optimization scheme to derive liquid densities from data collected in a pink X-ray beam. We present the results of the outlined procedure on two datasets: a simulated pink beam diffraction spectrum derived using the radial distribution function from a quantum molecular dynamics (QMD) simulation, and an experimental spectrum recorded at the dynamic compression sector for liquid tin. The densities derived for the experimental data are compared with other experimental studies and the Sesame 2161 EOS table. We conclude the paper by providing a brief summary and some practical considerations while using the proposed scheme.

## 2. Methods

This section mathematically describes the main ideas of this work. The section proceeds in the following steps: a brief overview of the coherent diffraction intensity for a monatomic liquid using the Debye scattering equation is presented. Next, following the work presented by Bratos *et al.* (2014 ▸), the modulation of this diffracted intensity in the presence of a pink X-ray beam is outlined. The section describes the pink beam correction using a Taylor series to approximate the change in diffraction intensity as a function of momentum transfer. Finally, the density optimization algorithm using the pink beam correction is presented.

### 2.1. Monatomic liquids

X-ray scattering from a disordered group of *N* atoms is given by the Debye scattering equation as (Debye, 1915 ▸) 

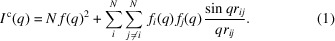

Here, *r*_*ij*_ is the distance between atoms (*i*, *j*), *q* is the momentum transfer and *f* is the atomic form factor. For monatomic liquids, the structure factor, *S*(*q*), is related to the experimentally observable coherent diffraction intensity, *I* ^c^ (ignoring central scattering), by 

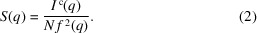

The structure factor is related to the radial distribution function, *g*(*r*), by the following equation, 

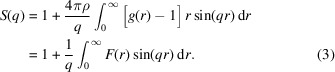

Here, *F*(*r*) = 4πρ[*g*(*r*) − 1]*r* and ρ is the mean density of the liquid. Readers are referred to Warren (1990 ▸) for a detailed overview of scattering by liquids.

### 2.2. Structure factor from experimental scattering intensities

The experimental diffraction signal recorded from liquids can be converted to the structure factor, *S*(*q*), by computing a normalization factor. The normalization factor, α, is computed as the value which minimizes the quantity 

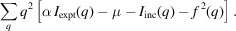

Here, *I*_expt_ denotes the experimentally recorded diffraction intensity, μ refers to multiple scattering, *I*_inc_ denotes the theoretically calculated incoherent intensity and *f* ^2^ refers to the the squared atomic scattering factor of the liquid. μ is independent of *q* and is another free variable along with α in the optimization problem. The theoretical incoherent scattering and atomic form factors for different atoms have been tabulated as a function of the scattering parameter, *s* = *q*/4π (Smith *et al.*, 1975 ▸; Brown *et al.*, 2006 ▸). The structure factor is obtained from the experimental diffraction intensity using the following formula, 

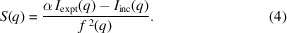

All symbols have been previously defined. The readers are referred to the classic papers of Ashcroft & Langreth for more details (Ashcroft & Langreth, 1967*a* ▸,*b* ▸, 1968 ▸). The structure factor obtained after this is then used in an iterative loop to reduce the oscillations at small atomic distances, *r*, below the first interatomic peak of the monatomic liquid. This iteration typically converges in a few steps. The readers are referred to Eggert *et al.* (2002 ▸) for further details about this iterative procedure.

### 2.3. Signal modulation by pink X-ray beam

The coherent scattering signal from a liquid is modified in the presence of a pink X-ray beam. This is given by a weighted sum of the scattering by the liquid for the different energies, *E*′, in the pink beam (Warren, 1990 ▸; Bratos *et al.*, 2014 ▸). The weights, *w*, as well as the limits of the integration, *E*_min_ and *E*_max_, are given by the energy spectrum produced by the undulator. Mathematically, 

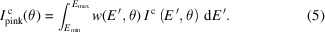

Here, *I* ^c^ denotes the coherent diffraction intensity. Instead of using the variable θ, it is useful to transform equation (5) in terms of the momentum transfer, *q*. Let *E* ^*M*^ be some energy in the pink spectrum with non-zero photon flux. The pink coherent scattering intensity, 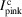 , as a function of the scattering angle θ can be transformed to an equivalent scattering intensity as a function of the momentum transfer variable *q* ^*M*^, where 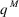 = 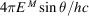 , where *h* and *c* are Planck’s constant and the speed of light, respectively. Equation (5) transforms to 

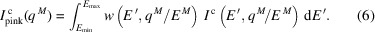

Since the weights, *w*, are only dependent on the energy, its dependence on θ through the variable *Q* ^*M*^/*E* ^*M*^ will be dropped for all subsequent equations. An obvious choice for *E* ^*M*^ would be the photon energy with the highest flux, but there is no *a priori* reason for this. As we shall see later, we treat this energy as another variable to be determined during density optimization.

### 2.4. Pink beam correction

As discussed in Section 2.4, the liquid diffraction signal recorded in a pink X-ray beam is a linear combination of the diffraction signal resulting from each energy in the X-ray. Therefore, the usual analysis methods used for monochromatic X-ray beams cannot be employed directly. However, if the pink beam has a narrow energy bandwidth, as is the case at the Dynamic Compression Sector, the pink beam diffraction spectra scan be corrected to an equivalent quasi-monochromatic diffraction signal. Once this correction is performed, the known analysis methods for monochromatic liquid diffraction spectra are valid. This section outlines the procedure for performing this correction. The change in the scattering signal as a function of *q* can be approximated by a Taylor series as 

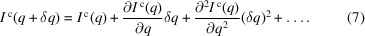

In the limit 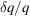 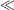 1, this expression is well approximated by the first-order term. For the U17 undulator spectrum at DCS, the flux decreases by a factor of 1/e over the energy range Δ*E* ≃ 0.88 keV. This corresponds to δ*q*/*q* ≃ 0.037, significantly smaller compared with 1. The pink beam sources at other synchrotron sources, such as the European Synchrotron Radiation Facility (ESRF), are sharper compared with DCS (δ*q*/*q* ≃ 0.024) (Wulff *et al.*, 2003 ▸). We will make this assumption for the rest of this manuscript. Substituting the expression for *I* ^c^(*q*) from equation (2), the derivative of the coherent scattering intensity is given by

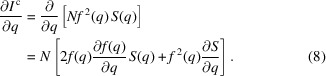

Using the expression for the liquid structure factor from equation (3) results in the following expression for the derivative of the structure factor with respect to *q*, 

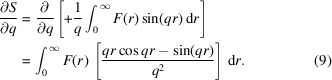

Using the expression for the derivative of the scattering factor in equation (9) and replacing it in equation (8) results in the following expression for the derivative of the coherent scattering intensity,

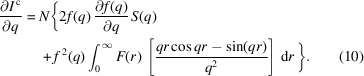

The form factors, *f*(*q*), in the previous equations are tabulated for each atom as a function of the parameter *s* = *q*/4π. The form factors are expressed as a weighted sum of Gaussians of different widths and a constants term. Since these functions are smooth, the derivatives of the form factors are trivial to compute as well. The expression is presented in the following equations. The values of *A*_*i*_, *B*_*i*_ for different atoms have been tabulated and can be found in the *International Tables of Crystallography* (Brown *et al.*, 2006 ▸),

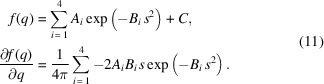

The derivative of the coherent scattering intensity with respect to the momentum transfer can be converted to the derivative with respect to the photon energy *E* by using the chain rule, 

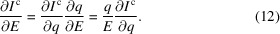

Substituting the above expression in equation (10) results in the following equation for the derivative of the scattering intensity with the photon energy, 

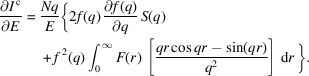

The expression for the derivative presented in the previous equation can now be plugged into equation (5) to compute the modulation of the scattered X-rays as a result of the pink X-ray beam. This is given by 

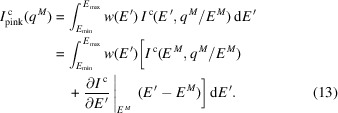

Notice that *I* ^c^(*E* ^*M*^, *q* ^*M*^/*E* ^*M*^) and 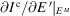 are independent of the integration variable. This allows us to write the above equation as 

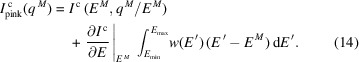

The integral can be computed with the knowledge of the pink beam spectra. Note that, for symmetric undulator spectra, this integral goes to zero. Therefore, in the first-order approximation, the correction term goes to zero for a symmetric profile. However, this is not true if higher terms are included in the Taylor series. The effective monochromatic spectra can be computed by subtracting the correction term from the pink beam spectra. The effective monochromatic spectrum is given by 

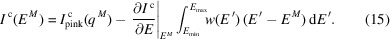

The above equation is only valid for a monatomic liquid. A similar correction for polyatomic liquid can also be calculated using the formalism described above but is beyond the scope of this work.

### 2.5. Termination function

To partially eliminate effects of limited *q*-range in the measured signal, the termination function described by Kuwayama *et al.* (2020 ▸) was used. This method extends the structure factors beyond the recorded limit, *q*_max_, using the following equation, 

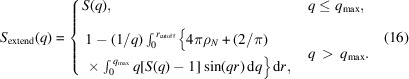

Here, ρ_*N*_ refers to the number density. The other symbols have previously been defined in the text.

### 2.6. Density optimization

The above formalism assumes the knowledge of *F*(*r*) to compute the derivative of the coherent scattering intensity. However, this information is not known *a priori* and needs to be extracted from the measured diffraction intensity. Therefore, a bootstrap method was used. This is outlined in the algorithm below:

(1) Compute *g*(*r*) [inverse Fourier transform of equation (3)] without any correction and assuming a monochromatic spectrum using the measured signal.

(2) Compute correction factor using equation (13) and *g*(*r*) from step 1.

(3) Use correction factor from step (2), correct for pink beam using equation (15) and recompute *g*(*r*).

(4) Use the corrected *g*(*r*) to update the current values for the input parameters.

(5) Repeat steps (2)–(4) until converged.

It should be noted that, due to the limited *q*-range of the measured signal, iterative application of the correction can lead to a growing fluctuation in *S*(*q*). This is related to the rapidly oscillating nature of the derivative of the sinc function [second term on the right in equation (10)]. Practically, only one iteration leads to acceptable results and avoids numerical instability. The corrected structure factor is then fed into a similar optimization procedure to the one outlined by Eggert *et al.* (2002 ▸) to extract the density. The optimization problem seeks to minimize the following function, 

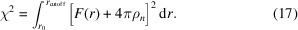

**Figure 2:**
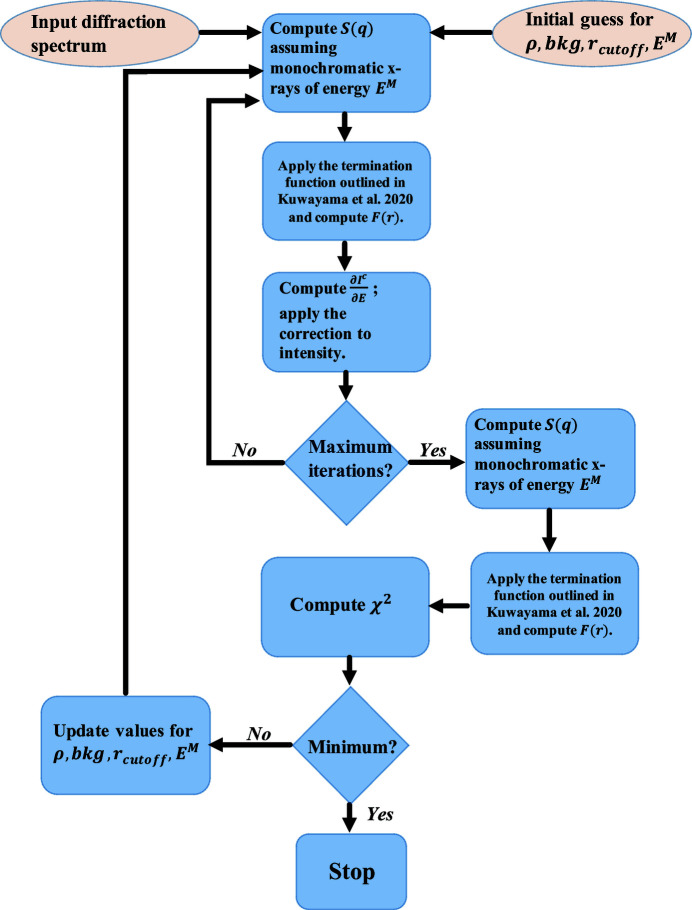
Density optimization algorithm. Symbols have been defined in the text.

Here, ρ_*n*_ defines the number density in atoms/Å^3^. The optimization is performed over four variables, namely ρ_*n*_, bkg, *r*_cutoff_ and *E* ^*M*^, where bkg is the constant background signal, *r*_cutoff_ denotes the interatomic distance below which the radial distribution function should vanish, and *E* ^*M*^, defined in Section 2.3, denotes the energy in the Taylor series approximation. Fig. 2 ▸ outlines the density optimization algorithm. The optimization parameters are updated using the BOBYQA optimization algorithm (Powell, 2009 ▸; Cartis *et al.*, 2019 ▸). Note that the algorithm accepts bounds constraints on variables. In our experience, constraints of ±0.2–0.3 Å for *r*_cutoff_ around an initial guess derived from QMD simulations and ±1 keV around an initial guess of the peak flux energy for *E* ^*M*^ are a good choice.

### 2.7. Uncertainty estimation

The main contribution to the uncertainty in liquid density obtained from this analysis originates from the residual errors after correcting for the non-monochromatic X-ray source. Using simulated liquid diffraction intensities derived from QMD trajectory simulations of high *P*–*T* liquid Sn, it was estimated that the error in density is ∼0.75% when diffraction intensity is available up to 12 Å^−1^. The errors increase to ∼2% when the intensities are only available up to 8 Å^−1^. The *q*-range at DCS is closer to the latter case. The estimation was done by performing thousands of optimizations for different initial guesses for the four optimization parameters and calculating the distribution of the derived densities. This uncertainty is a systematic error, with the final estimate from the analysis always lower than the true value resulting in asymmetric lower and upper uncertainty bounds (the upper bound is larger). The second source of uncertainty arises from the spread in the final converged value of density for a number of randomly chosen initial guesses. Therefore, density optimization is started from a number of initial guesses and the standard deviation of the final converged values is added to the systematic uncertainty. Finally, uncertainty in the geometric calibration of the X-ray detector also leads to some uncertainty in the derived density. This value is much smaller than the other two values and will be ignored for this study. The total uncertainty in density is a sum of the individual contributions. The method described here has been employed for experimental silver data, applying uncertainty bounds to coordination number determined from density measurements in shock compressed liquid states up to 330 GPa (Coleman *et al.*, 2022 ▸).

## 3. Results

This section presents results of the outlined procedure for two different cases: a simulated pink beam diffraction signal derived from QMD simulation and an experimental diffraction signal recorded at the Dynamic Compression Sector. The simulated results have a known ground truth and provide a good measure for the efficacy of the proposed method. The density derived from the experimental diffraction signal is compared with densities derived from other shock compression and gas gun studies as well as the Sesame 2161 pressure–density table.

### 3.1. QMD simulations – tin

QMD simulations based on density functional theory were performed for liquid Sn using a cubic cell with 128 atoms. We use the Baldereschi *k* point (Baldereschi, 1973 ▸) of (1/4; 1/4; 1/4)2π/*a* to sample the Brillouin zone. Here, *a* is the side length of the simulation cell. We choose the Perdew–Burke–Ernzerhof for solids (PBEsol) exchange-correlation functional (Blöchl *et al.*, 1994 ▸), 400 eV cutoff for the plane wave basis, and a projected augmented wave (PAW) pseudopotential that has a core of 3.0 Bohr and treats 5*s* ^2^5*p* ^2^ as valence electrons as provided in the Vienna Ab-Initio Simulation Package (VASP) (Kresse & Furthmüller, 1996 ▸). A Nosé thermostat was used to generate MD trajectories in a canonical (NVT, *i.e.* constant number of atoms, constant volume, and constant temperature) ensemble. The MD trajectory consisted of 12000 steps with a time step of approximately 1.5 fs. The radial distribution function is calculated by analyzing interatomic distances along the MD trajectory after the system reaches equilibrium, from which the structure factor is calculated following the procedure outlined by Zhang & Morales (2020 ▸). We note that the simulation being reported here corresponds to a temperature of 5000 K. We have performed additional calculations at ±1000 K, using different cell sizes (up to 256 atoms), finite *k* mesh, and other exchange-correlation functionals, and found similar results for the calculated pressure and *g*(*r*) profile.

**Figure 3:**
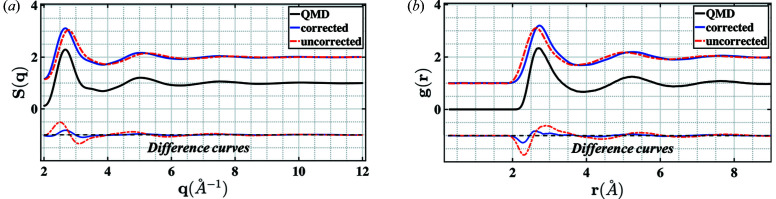
(*a*) Structure factor and (*b*) radial distribution function for tin derived from QMD simulations at 11.0 g/cc and 5000 K. The finite size of the QMD simulation cell results in structure factors below ∼2 Å^−1^ being unreliable. The difference curve of the corrected and uncorrected curves with the QMD results is shown as well.

The radial distribution function was extracted from QMD simulations, and equations (2), (3) and (5) were used to generate the ‘experimental’ diffraction signal for pink X-ray beam. The energy–flux distribution recorded for the U17 undulator at DCS as shown in Fig. 1 ▸ was used for this calculation. The procedure outlined in this work was then used to extract the structure factor, radial distribution function and mean density. This is represented by the ‘corrected’ curved in Figs. 3 ▸(*a*) and 3(*b*). The ‘uncorrected’ curves do not account for the non-monochromatic source and assumes that the coherent diffraction signal was recorded at a monochromatic source with X-ray energy corresponding to the energy with the peak flux in Fig. 1 ▸ (red curve ∼23.53 keV). This data set lets us benchmark the algorithm for the ideal case where the mean density is already known.

The theoretical density (in g cm^−3^) along with the densities obtained for the uncorrected and corrected cases are listed in Table 1 ▸. *r*_cutoff_ and *E* ^*M*^ converged to the physically reasonable values of 2 Å and 23.48 keV, respectively. The uncertainties were estimated using the method described in Section 2.8. The starting density was uniformly sampled in the interval 10.5–11.5 g cm^−3^. The uncertainties reported in Table 1 ▸ are the standard deviation for 100 different starting values. It is interesting to note that the uncertainties for the corrected density are not large enough to account for the deviation of the estimated density with the theoretical value. This indicates that, although the approximation clearly improves the estimate, the Taylor series approximation is not exact and introduces a small (<1%) error in the final estimate. This is likely a result of the first-order approximation made in this work. However, including higher-order terms is infeasible due to the limited *q*-range in experimental data. This introduces unwanted fluctuations in the corrected structure factors and leads to erroneous estimates of density. The QMD results for the structure factor and radial distribution functions, along with the uncorrected and corrected spectra for these quantities, are presented in Fig. 3 ▸.

### 3.2. Experiment – tin

Laser shock compression experiments were carried out at the Dynamic Compression Sector of the Advanced Photon Source synchrotron, Argonne National Laboratory (Wang *et al.*, 2019 ▸). Shock targets consisted of 50 µm of polyimide ablator, glued to 29.5 µm Sn foils (99.75%, Goodfellow). A 500 µm-thick single-crystal LiF window was glued to the rear surface of the target package and velocimetry measurements were collected at the Sn/LiF interface using a point VISAR system. Pressure was determined by impedance matching the measured Sn/LiF particle velocity and comparing with the Sn EOS (Sesame 2161) (Greeff *et al.*, 2005 ▸).

X-ray images were collected on a Rayonix SX165 area detector (2048 × 2048 pixels) and timed with respect to the laser pulse such that diffraction was collected just before the shock wave reaches the Sn/LiF interface. Consequently, a small portion of ambient diffraction from uncompressed Sn ahead of the shock wave is masked from diffraction profiles so that only liquid scattering signal is considered for analysis. Single-crystal Laue diffraction spots from the single-crystal LiF window are also masked. Finally, the detector response is removed from the shocked images before azimuthally integrating using Dioptas (Prescher & Prakapenka, 2015 ▸). This approach was shown to work well for data collected at the LCLS (Briggs *et al.*, 2019*a* ▸) and at DCS (Briggs *et al.*, 2019*b* ▸). However, the contribution of background signal from the plastic ablator will be more significant for lower-*Z* materials and would require additional background subtraction, similar to the removal of empty cell background as described by Eggert *et al.* (2002 ▸). In this work the ratio of the squares of atomic numbers, representing the scattering cross-section for Sn and Kapton (CH), is ∼100, and the contribution of the 50 µm plastic ablator is negligible (we observe no amorphous or crystalline signal from the plastic ablator) at ∼23.5 keV.

**Figure 4:**
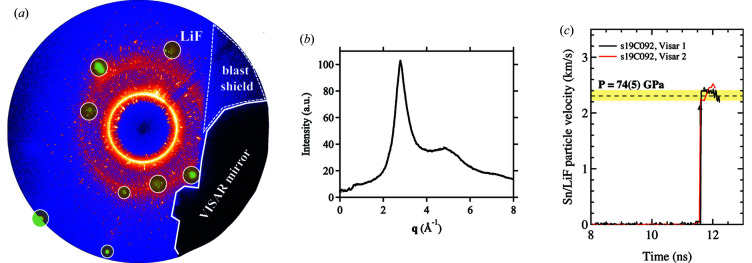
(*a*) Raw diffraction profile collected at DCS. Single-crystal spots from the LiF window material, which are masked from the integrated profiles, are highlighted by green circles. All remaining spots are ambient β-Sn peaks (ambient material ahead of the shock wave) that are also removed from the final integrated profile, leaving only the diffuse scattering from liquid tin. The X-ray shadows from the VISAR mirror and blast shield are also highlighted. (*b*) Partial azimuthally integrated intensity as a function of momentum transfer from (*a*) ignoring the areas with shadows from the blast shield and the VISAR mirror. The beam energy used for converting from 2θ to **q** was 23.53 keV. (*c*) Point VISAR data. The dashed line shows the average Sn/LiF particle velocity at shock breakout, with uncertainty bounded by the yellow shaded region; the shock pressure of 74 (5) GPa is determined using impedance matching of the Sn sample with the LiF window. The arrow indicates the time at which there is a significant loss of reflectivity.

Consequently, a small portion of ambient diffraction from uncompressed Sn ahead of the shock wave is masked from diffraction profiles so that only liquid scattering signal is considered for analysis. Single-crystal Laue diffraction spots from the single-crystal LiF window are also masked. The readers are referred to Briggs *et al.* (2019*a* ▸) for details about the background removal procedure. See Fig. 4 ▸(*a*). The detector response was removed from the shocked images, before azimuthally integrating using *Dioptas* (Prescher & Prakapenka, 2015 ▸).

The sample detector distance, rotation, and tilt were calibrated using the diffraction lines of polycrystalline Si and cross-checked with CeO_2_ (NIST). The azimuthally integrated intensity after removing the diffraction from crystalline Sn and LiF window as well as the shadowed regions from the blast shield and the VISAR mirror is shown in Fig. 4 ▸(*b*). A pressure of 74 (5) GPa was estimated from VISAR as shown in Fig. 4 ▸(*c*). Similar experiments were performed on the Matter in Extreme Conditions (MEC) instrument using the monochromatic X-ray Free Electron Laser at the Linac Coherent Light Source (LCLS-II) to achieve similar densities. The results have been previously reported by Briggs *et al.* (2019*a* ▸). The study reported a mean density of 11.2 (1) g cm^−3^. This corresponds to an estimated pressure of 79 (8) GPa on the Sesame 2161 Hugoniot. Earlier work using multi-anvil apparatus studied liquid tin only up to 20 GPa (Narushima *et al.*, 2007 ▸) at temperatures just above the melting curve (Briggs *et al.*, 2017*b* ▸).

**Figure 5:**
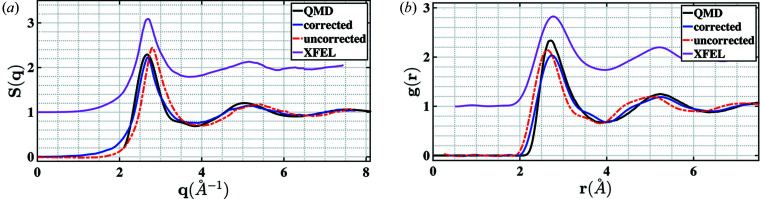
(*a*) Structure factor and (*b*) radial distribution function from shock melted tin for measurements at LCLS-II, DCS (corrected and uncorrected) as well as the QMD simulations. While a direct comparison is not possible (see text), the proposed algorithm leads to reasonable results for liquid tin of comparable pressures and densities.

Density optimization without accounting for the pink beam resulted in a mean density of 13.67 (7) g cm^−3^. Accounting for the pink beam effect resulted in a density of 11.0 (2) g cm^−3^. The QMD simulations from the previous example in Section 3.1 had a pressure of 80 GPa, comparable with the value estimated from VISAR analysis. The structure factors and the radial distribution functions obtained using the data recorded at DCS and MEC along with the QMD simulations are presented in Fig. 5 ▸. Since the temperatures are not measured during experiments, the precise thermodynamic state of the melted tin is not known. This makes the direct comparison between the two experimental measurements and the QMD simulations difficult. The purpose of presenting these datasets is not to perform a quantitative comparison but to demonstrate that the algorithm outlined in this work leads to reasonable estimates of the radial distribution functions for liquid tin of comparable densities. The coordination number of the corrected spectra, related to the area under the first *g*(*r*) peak, shows agreement with the QMD simulations and the MEC data once the correction is applied. The coordination number (CN) was determined using the following equation (Morard *et al.*, 2014 ▸),

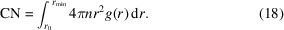

Here, *n* is the number density and *r*_0_ and *r*_min_ are the integration limits corresponding to the left edge of the first peak and the first minima in *g*(*r*), respectively. There are competing methods prescribed to evaluate *r*_min_, where *r*_min_ corresponds to the first minima of the function 4π*r* ^2^ *g*(*r*) (Waseda, 1980 ▸). This convention was used by Briggs *et al.* (2019*a* ▸) to compute the coordination number. Table 2 ▸ lists the coordination number obtained by using the definition of *r*_min_ in Morard *et al.* (2014 ▸) (method I) and Waseda (1980 ▸) (method II). The values indicate that using the same cutoff value for *r*_min_ leads to consistent results.

Due to the higher density estimate, the uncorrected radial distribution function overestimates the coordination number significantly. Zhang & Morales (2020 ▸) used method II to compute the CN and argue that, since the body-centered-cubic phase of tin has 8 first nearest neighbors and 6 second nearest neighbors, a coordination number close to 14 is reasonable for the liquid phase, and indicates that these two shells merge into one. These results are summarized in Table 2 ▸. The uncertainty in the mean density for both the uncorrected and corrected spectra is estimated by initializing the optimization at 100 uniformly sampled density values in the interval 10.5–11.1 g cm^−3^.

**Figure 6:**
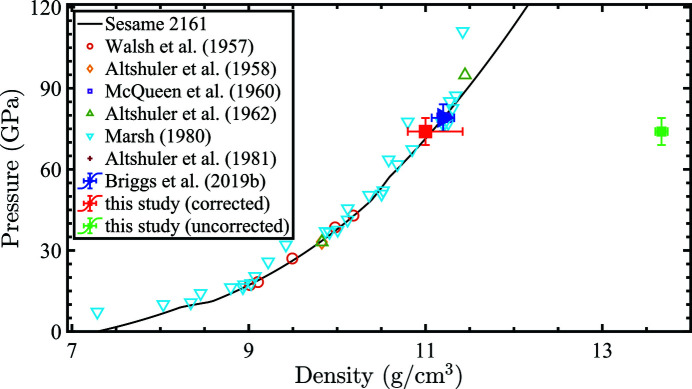
Tin densities on the Hugoniot derived from gas gun experiments (Walsh *et al.*, 1957 ▸; Altshuler *et al.*, 1958 ▸; McQueen & Marsh, 1960 ▸; Altshuler *et al.*, 1962 ▸, 1981 ▸; Marsh, 1980 ▸) with density derived from the current study plotted with the red glyph. The SESAME 2161 pressure–density table is shown with the solid black line (Greeff *et al.*, 2005 ▸). The density without accounting for the pink beam is shown by the green glyph. It should be noted that the reported liquid density using dynamic compression experiments in Briggs *et al.* (2019*a* ▸) (blue glyph) was estimated by pinning the VISAR pressure on the Sesame 2161 hugoniot. Quantitative estimate was not possible using the collected diffraction signal.

To put our density estimates into a broader context, the estimated densities were compared with other experimental data points as well as the tabulated Sesame 2161 Hugoniot. This has been presented in Fig. 6 ▸. The density estimate, after applying the pink beam correction, agrees very well with previously measured data as well as the Sesame 2161 tables for tin. The density estimate without accounting for the pink beam artifacts, shown by the green glyph, is extremely poor and shows the significant impact of the pink beam on the density estimates.

## 4. Discussion and conclusions

In this paper, we have outlined a new methodology to correct for the artifacts introduced in the diffraction signal produced by liquids in a pink X-ray beam. The correction relies on the first-order Taylor’s series expansion of the coherent diffraction intensity. The proposed method is bench-marked with simulated tin data of known density and temperature. The method was able to correct for the pink beam effect to less than 1% error. Finally, the method was demonstrated for an experimental liquid scattering signal from tin recorded at the Dynamic Compression Sector. The mean density and radial distribution function compare favorably with both experimental results recorded with a monochromatic source at the Linac Coherent Light Source as well as QMD simulations. The density estimates after applying the correction are in excellent agreement with other experimentally recorded data as well as the Sesame 2161 tables for tin.

The above treatment is a practical one as there are no theoretical guarantees that the algorithm will converge. However, in most scenarios the methodology is able to correct for the measurement artifacts introduced by the pink X-ray beam. Furthermore, introduction of new variables into the optimization problem makes it harder to find the global minima. Care must be taken in choosing the initial starting values and the bounds specified. QMD can be used to guide the starting values and expected ranges of these parameters. Alternatively, a more robust global optimization algorithm can be utilized. This comes at an increased computational cost. Finally, a limited *q*-range in the measured data can introduce unwanted fluctuations in the corrected structure factor and radial distribution function. Therefore, the results need to be evaluated with caution to ensure that such a fluctuation is not interpreted as a true feature in the data.

## Figures and Tables

**Table 1:** Theoretical mean density and densities obtained by the outlined optimization procedure before and after correcting for the pink beam effect Percentage errors from the theoretical value are given in parentheses. All densities are in units of g cm^−3^.

Theoretical density	Uncorrected density	Corrected density
11.0	12.250 (2)	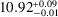
	(6.90%)	(0.73%)

**Table 2:** Densities and coordination numbers for liquid tin from different datasets Two different methods used for calculating the CN, as discussed in Section 3.2, have been presented as method I and II.

Data source	Pressure (GPa)	Density (g cm^−3^)	CN (method I)	CN (method II)
DCS (uncorrected)	74 (5)	13.67 (7)	15.5 (1)	13.2 (1)
DCS (corrected)	74 (5)	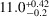	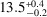	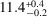
XFEL @ LCLS-II	79 (8)	11.2 (1)	13.7 (1)	11.1 (1)
QMD simulations	79.95 (8)	11.0	13.6	12.0
